# Combined Effects of Nasal Ketamine and Trauma-Focused Psychotherapy in Treatment-Resistant Post-Traumatic Stress Disorder: A Pilot Case Series

**DOI:** 10.3390/bs14080717

**Published:** 2024-08-16

**Authors:** Judith Rohde, Elena Hickmann, Marco Buchmann, Golo Kronenberg, Stefan Vetter, Erich Seifritz, Birgit Kleim, Sebastian Olbrich

**Affiliations:** 1 Department of Adult Psychiatry and Psychotherapy, Psychiatric University Hospital Zurich, Lenggstrasse 31, CH-8032 Zurich, Switzerland; elena.hickmann@pukzh.ch (E.H.); golo.kronenberg@pukzh.ch (G.K.); stefan.vetter@pukzh.ch (S.V.); erich.seifritz@bli.uzh.ch (E.S.); birgit.kleim@pukzh.ch (B.K.); sebastian.olbrich@pukzh.ch (S.O.); 2 Department of Psychology, University of Zurich, Binzmuehlestrasse 14, CH-8050 Zurich, Switzerland; marcopietro.buchmann@uzh.ch

**Keywords:** ketamine, ketamine assisted, PTSD, trauma focused, psychotherapy

## Abstract

Objective: This pilot case series investigated the feasibility and efficacy of an eight-week therapy program, combining nasally administered ketamine (0.5 mg/kg) with trauma-focused psychotherapy, for individuals with chronic, treatment-resistant post-traumatic stress disorder (PTSD). Method: Three patients with chronic, treatment-resistant PTSD underwent the eight-week therapy program. Clinical assessments included the Clinician-Administered PTSD Scale for DSM-5 (CAPS-5) and the Hamilton Depression Rating Scale (HAMD) at baseline, post-assessment, and follow-up assessment, along with additional measures assessing other relevant symptoms and side effects. Results: The results demonstrated clinically relevant reductions in PTSD symptoms, indicated by a change in the CAPS-5 score at post-assessment (*M* = −18.00; *SE* = 6.48) and follow-up assessment (*M* = −25.33, *SE* = 5.58). Additionally, depressive symptoms showed notable improvement, with changes in HAMD scores at post-assessment (*M* = −8.33, *SE* = 3.07) and follow-up assessment (*M* = −9.00, *SE* = 3.77). Positive effects were also observed in anxiety reduction, decreased dissociations, and improvements in emotion regulation and disturbances of self-organization. Conclusions: Despite potential variations in clinical profiles among the patients, the therapy program demonstrated positive outcomes for all participants. Nasally administered ketamine was well tolerated and resulted in immediate symptom reduction in tension, anxiety, and common PTSD symptoms. However, to validate these findings and compare treatment efficacy, future randomized controlled trials are warranted, especially in comparison with trauma-focused therapy alone.

## 1. Introduction

Post-traumatic stress disorder (PTSD) is a debilitating disorder and affects 4% of the global population [1]. Trauma-focused psychotherapy is recommended as a first-line treatment [2,3,4,5], while pharmacological options are rare and less efficient [6,7]. For instance, guidelines primarily recommend selective serotonin reuptake inhibitors (SSRIs), with only sertraline and paroxetine approved by Swissmedic, the EMA, and the FDA [8,9,10]. These SSRIs show modest efficacy in improving PTSD symptoms, with effect sizes lower than those observed in major depressive disorders [11]. Nevertheless, psychotherapeutic treatments, such as trauma-focused cognitive behavioral therapy, also exhibit limitations, including notable dropout rates [12,13] and insufficient symptom reduction for a significant group of patients [14,15]. Given these challenges, it is evident that existing treatments for PTSD are not universally effective, indicating a pressing need for further options in the therapeutic landscape.

(R,S)-Ketamine, hereafter referred to as ketamine, is well-known for its longstanding use in anesthesia and pain management [16]. The antidepressant properties of esketamine led to its approval by the FDA in the therapy of treatment-resistant depression in 2019, as well as approval by the EMA [16,17]. Recent studies highlight ketamine’s potential in reducing PTSD symptoms. Feder and colleagues demonstrated its efficacy through single and repeated intravenous doses of 0.5 mg/kg, showing rapid and safe symptom reduction within 24 h, but with limited duration [18,19]. In their 2014 study, the primary outcome measure was PTSD symptom severity assessed with the Impact of Event Scale-Revised (IES-R) 24 h after infusion, with a sample size of 41 patients. The mean difference in IES-R scores was 12.7 (95% CI, 2.5–22.8; *p* = 0.02) in crossover analysis and 8.6 (95% CI, 0.94–16.2; *p* = 0.03) in first-period analysis. In their 2021 study, the primary outcome measure was the change in PTSD symptom severity measured with the Clinician-Administered PTSD Scale for DSM-5 (CAPS-5), involving 30 participants, showing significant improvements in PTSD and depressive symptoms. Others found reduced symptoms lasting seven days post single ketamine infusion (0.5 mg/kg) in patients with chronic pain and PTSD [20], with the primary outcomes being PTSD symptom severity assessed with the IES-R and pain severity with the Visual Analogue Scale (VAS). The sample size was 41 participants, showing significant decreases in PTSD symptoms from baseline to one day post-infusion (*t*(32.59) = 2.33, *p* = 0.03) and from baseline to seven days post-infusion (*t*(27.53) = 2.93, *p* < 0.01). Combining a single ketamine dosage (0.5 mg/kg) with 12 sessions of TIMBER, a mindfulness-based cognitive behavioral therapy for trauma-related disorders, extended its effects [21]. The study involved 10 patients, with primary outcomes measured using the PTSD Checklist (PCL) and Clinician-Administered PTSD Scale for DSM-IV (CAPS-IV). Significant differences were observed in PCL scores between baseline and 24 h after infusion (*p* < 0.001) and in CAPS scores (*p* < 0.001). However, Abdallah and colleagues observed improvements in depression, not in PTSD, with eight ketamine doses (0.2 or 0.5 mg/kg) over four weeks [22]. The study included 158 participants, with primary outcomes measured using the PCL-5 and secondary measures measured using the CAPS-5 and Montgomery–Asberg Depression Rating Scale (MADRS). The effect sizes for the PCL-5 were 0.96 (standard dose), 0.93 (low dose), and 0.75 (placebo) 24 h post-first infusion.

Apart from randomized trial by Pradhan and colleagues, to our knowledge, only six other studies, comprising three case studies [23,24,25], one chart review [26], one open-label study [27], and a pilot study [28], investigated ketamine’s combination with psychotherapy. Ketamine application frequencies varied from one to six administrations, predominantly intravenously in three studies, sublingually in two, and intramuscularly in one. Nevertheless, detailed information regarding the timing of psychotherapy sessions in relation to ketamine administration and specific psychotherapy contents is inconsistently reported. While most studies conducted some psychotherapy sessions before, during, or immediately after ketamine administration, only two explored 24–36 h sessions post-ketamine application [23,27].

While doses ranging from 1 to 4.5 mg/kg are used for the induction of anesthesia, subanesthetic doses of 0.5 mg/kg (IV) are most commonly used for depression treatment [16]. Intranasal treatments with ketamine have been studied less frequently, but doses of 50 mg of ketamine for depression treatment have been reported [29]. Additionally, doses of 28–84 mg of esketamine, depending on the age, are recommended for the treatment of therapy-resistant depression [10]. No intranasal doses for the treatment of PTSD have been reported yet.

Trials examining ketamine’s effects on PTSD, especially in combination with state-of-the-art trauma-focused psychotherapy, remain limited. Moreover, none have investigated nasal administration. Persisting challenges include the absence of standardized treatment protocols and uncertainties regarding optimal administration routes as well as dosing regimens [30]. Given the need for innovative and efficacious treatments, we investigated the combination of trauma-focused therapy with repeated nasal ketamine (0.5 mg/kg) in chronic, treatment-resistant PTSD, aiming to assess its impact on trauma-related symptoms.

## 2. Methods

Three patients were referred to the outpatient clinics of Psychiatric University Hospital Zurich, presenting with chronic, severe PTSD and had undergone at least two unsuccessful disorder-specific treatments (psychotherapy and psychopharmacotherapy) in their medical history. Table 1 lists all inclusion and exclusion criteria. All participants consented to the publication of these results.

Following a psychological (Supplemental Table S1) and a physical assessment as part of clinical routine diagnostics, patients were enrolled in an eight-week therapy program with weekly ketamine administration and trauma-focused psychotherapy sessions the day after. Ketamine was administered nasally at a dosage of 0.5 mg/kg (with an accuracy of 5 mg), a subanesthetic dose, which was based on existing literature. During the ketamine sessions, patients were comfortably seated or reclined on a couch in a quiet environment, with medical personnel monitoring their condition. Blood pressure, pulse, and oxygenation were measured before and at 40 and 120 min after ketamine application. Side effects were assessed before and 2 h after each application on a scale ranging from 1 standing “not at all” to 10 “very much” (Supplementary Table S2). Trauma-focused therapy was administered 24 h after each ketamine session. Therapy began with an initial session to establish a hierarchy of traumatic events, guiding the subsequent seven sessions. Techniques included at least three sessions of trauma exposure, where patients were gradually exposed to trauma-related memories and cues in a safe environment. Cognitive restructuring helped patients identify and modify distorted thoughts related to their trauma. Additional techniques included grounding exercises to reduce dissociation and meaning-making exercises to help integrate traumatic experiences. These therapies were delivered as usual by the patients’ regular therapists. After one month, psychological parameters were re-assessed (Supplementary Table S1).

Participants were selected based on their chronic and treatment-resistant PTSD, with a history of at least two unsuccessful specific treatments. Due to the small sample size, the results are descriptive and not generalizable.

**Figure 1:**
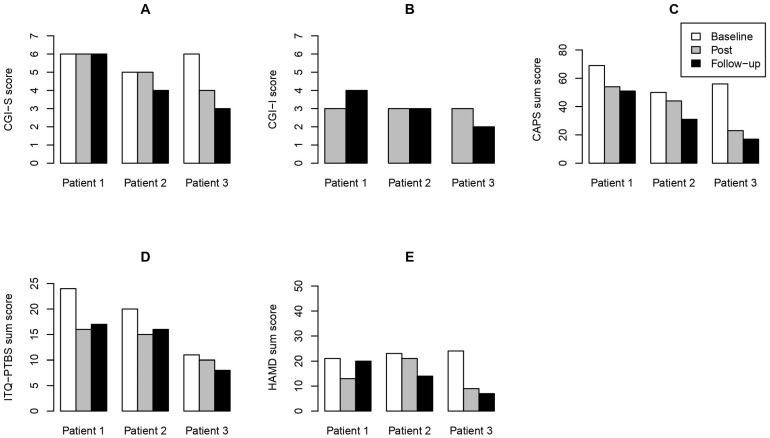
Main results. (**A**) Clinical Global Impressions Scale—Severity (CGI-S); (**B**) Clinical Global Impressions Scale—Improvement (CGI-I); (**C**) Clinician-Administered PTSD Scale for DSM-5 (CAPS-5); (**D**) International Trauma Questionnaire, PTSD subscale (ITQ-PTSD); (**E**) Hamilton Depression Rating Scale (HAMD); baseline = before 8-week treatment; post = after 8-week treatment; follow-up = one month after 8-week treatment.

All analyses and plots were created using R 4.4.1 [31]. All three participants were included in the analyses. From the psychological assessments (Supplementary Table S1), we used the sum scores and calculated the difference scores (mean reduction from baseline to post- and follow-up assessment, respectively) and standard error. All plots show individual sum scores except for Figure 1A,B, which display single item scores.

## 3. Results

Table 2 summarizes patients’ key socio-demographic and clinical characteristics. Blood test, physical examinations, and electrocardiograms revealed no significant abnormalities or contraindications to the administration of ketamine.

In the overall illness severity ratings assessed using the Clinical Global Impressions Scale—Severity, two out of three patients showed improvements at both assessment time points. Improvement ratings assessed using the Clinical Global Impressions Scale—Improvement showed ameliorations in all patients’ conditions at the post-assessment, with patients 2 and 3 maintaining these improvements at the follow-up assessment (see Figure 1A,B). All patients experienced clinically significant improvements in trauma-related symptoms with a clinician-rated reduction in PTSD symptom severity at post-assessment (*M* = −18.00; *SE* = 6.48) and follow-up assessment (*M* = −25.33, *SE* = 5.58) (Figure 1C) and with a self-rated reduction in PTSD symptom severity at post-assessment (*M* = −4.67, *SE* = 1.66) and follow-up assessment (*M* = −4.67, *SE* = 0.98) (Figure 1D). All patients showed an amelioration in depressive symptoms at post-assessment (*M* = −8.33, *SE* = 3.07) and follow-up assessment (*M* = −9.00, *SE* = 3.77) (Figure 1E).

**Figure 2:**
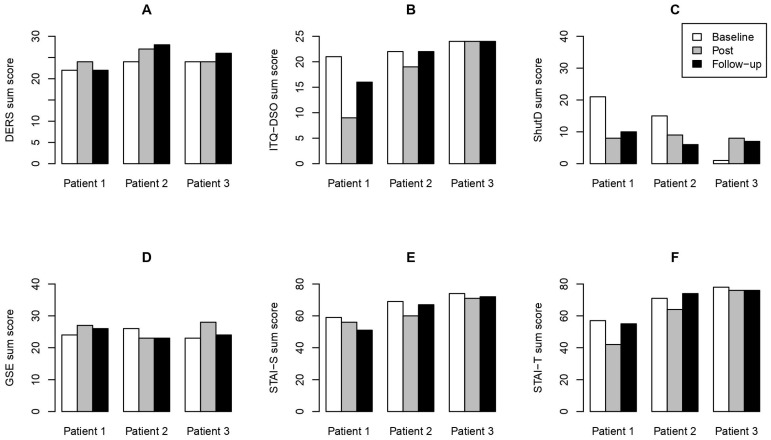
Additional results. (**A**) Difficulties in Emotion Regulation Scale (DERS); (**B**) International Trauma Questionnaire, DSO subscale (ITQ-DSO; (**C**) Shutdown Dissociation Scale (Shut-D); (**D**) General Self-Efficacy Scale (GSE); (**E**) The State-Trait Anxiety Inventory, state subscale (STAI-S); (**F**) The State-Trait Anxiety Inventory, trait subscale (STAI-T); baseline = before 8-week treatment; post = after 8-week treatment; follow-up = one month after 8-week treatment.

Additional findings reveal, among others (for all additional findings, see Figure 2), enhancements in emotion regulation for all patients, with the most noticeable improvement observed in patient 1 (Figure 2A). Patient 1 reported a decrease in disturbances in self-organization, while patients 2 and 3 either showed improvement only at the post-assessment or did not improve (Figure 2B). Dissociations were reported to occur less frequently in patients 1 and 2 and more frequently in patient 3 (Figure 2C; Complete data can be found in the Supplementary Materials (Supplementary Table S3).

Regarding side effects and other immediate effects 2 h after vs. before ketamine administration, the greatest changes were found (in descending order) in tension (*M* = −3.75, *SE* = 0.50), anxiety (*M* = −3.63, *SE* = 0.57), restlessness (M = −3.38, *SE* = 0.42), intrusions (M = −2.67, *SE* = 0.50), weak and warm body feeling (*M* = 1.38, *SE* = 0.43), and headache (*M* = −1.21, *SE* = 0.27). Complete data can be found in the supplementary materials (Supplementary Table S2). No severe side effects occurred. No hypertension crisis occurred (highest blood pressure was measured at 40 min after ketamine administration in patient 1 and was 163/97 mmHg). All patients completed the therapy program.

## 4. Discussion

We examined three patients suffering from chronic, therapy-resistant PTSD. We implemented an eight-week therapy program for the first time to the knowledge of the authors combining repeated nasally administered ketamine with trauma-focused psychotherapy. Our findings demonstrate that this approach resulted in clinically relevant reductions in PTSD and depressive symptoms. Additionally, we observed decreases in anxiety and dissociation, along with other notable improvements.

The clinical profiles of the patients were heterogeneous, which may account for some of the observed differences in treatment effects. Patient 1 had the highest symptom severity among participants and met the criteria for complex PTSD according to ICD-11 [32]. This patient showed improvements in PTSD symptoms, aligning with the literature on trauma-focused interventions, but also experienced notable enhancements in emotion regulation and a decrease in disturbances in self-organization (DSO), suggesting that the therapy program positively affects symptoms of complex PTSD, for which in fact multicomponent interventions would be the primary treatment option [33,34]. The increase in dissociations reported by patient 3 could potentially be attributed to the patient’s experiences following ketamine administration, as it corresponds with the ketamine side effects reported by this specific patient. Remarkably, the patients included, despite their history of unsuccessful PTSD treatments, were able to initiate and successfully complete trauma-focused treatment for the first time. This achievement may be associated with ketamine’s impact on reducing avoidance behavior [35].

Ketamine was well tolerated by all patients, and they benefited from immediate positive effects, which led to a decrease in tension, anxiety, intrusions, and other common symptoms associated with PTSD, while dissociations slightly increased. The observed reduction in anxiety is in line with the existing literature [36]. The immediate reduction in intrusions during and the overall positive impact on PTSD symptoms after the treatment can be attributed to ketamine’s role as an NMDA receptor antagonist, as the activation of this receptor is linked to an increase in intrusive memories. Moreover, ketamine normalizes decreased BDNF levels in the hippocampus, a pivotal structure for learning and memory [37]. In addition, studies have shown that ketamine increases synaptic plasticity and reverses both behavioral and neuronal changes that occur in the context of chronic stress [35,38,39].

To our knowledge, only a limited number of studies have investigated the combination of ketamine and psychotherapy in the treatment of PTDS, and most importantly, none of them have investigated nasal ketamine administration.

However, our case series has limitations, including a small sample size and lack of a control group or blinding. The observed reductions in symptoms are descriptive and cannot be generalized without further studies with larger sample sizes. We cannot distinguish which effects are attributable to ketamine or the psychotherapeutic treatment, since trauma-focused therapy, on its own, is an effective PTSD treatment, or their unique combination. Taken together, we showed clinically relevant improvements in PTSD symptoms in a cohort with chronic PTSD and unsuccessful prior treatments. The case series’ strength lies in pioneering an eight-week program with ketamine-assisted therapy, administered nasally, contributing to the quest for innovative PTSD treatments. Further research in a larger controlled study is essential to validate these encouraging findings.

## 5. Conclusions

This pilot case series indicates that combining nasally administered ketamine with trauma-focused psychotherapy can lead to significant reductions in PTSD and depressive symptoms in patients with chronic, treatment-resistant PTSD. The therapy was well-tolerated, and improvements were observed across several domains, suggesting that this combined approach may be effective for such challenging cases..

## Figures and Tables

**Table 1:** Inclusion and exclusion criteria.

Inclusion Criteria	Exclusion Criteria
18–65 years	Known aneurysmal vascular disease
Chronic PTSD (>6 months)	Known history of intracerebral hemorrhage
Two or more unsuccessful previous PTSD-specific treatments	Recent (within the last six weeks) cardiovascular event
	Known hypersensitivity to ketamine or esketamine
	Untreated hypertension
	Untreated liver, kidney, or lung disease
	Untreated hyperthyroidism
	History of traumatic brain injury of at least moderate severity
	History of (hypo-)manic or psychotic episodes
	Current use of opioids, benzodiazepines, or opioid antagonists
	Meeting criteria for a substance use disorder in the past 6 months
	Use of ketamine without medical prescription within the last two years
	Women of childbearing potential without effective contraception
	Current pregnancy
	If psychotropic medications are being taken: no stable medication including dosage within the last two months

**Table 2:** Socio-demographic and clinical characteristics.

	Patient 1	Patient 2	Patient 3
Age (years)	41	26	59
Sex	f	f	f
Diagnoses according to DSM-5	▪ Post-traumatic stress disorder * ▪ Dissociative disorder ▪ Attention deficit hyperactivity disorder (ADHD) ▪ Other specified feeding or eating disorder	▪ Post-traumatic stress disorder ▪ Major depressive disorder	▪ Posttraumatic stress disorder ▪ Major depressive disorder
Physical diseases	Mild mitral insufficiency	Migraine	None
Resting blood pressure (mmHg)	123/66	101/80	140/85
Number of stressful events personally witnessed (according to LEC-5)	8	3	3
CTQ score at baseline	77	70	67
CGI-S score at baseline	6	5	6
CAPS score at baseline	69	50	56
Concomitant psychotropic medication (per day)	▪ Methyphenidate 72 mg ▪ Chlorprothixene 15 mg	▪ Bupropione 300 mg ▪ Fluoxetine 20 mg	▪ Agomelatine 25 mg
Dosage of ketamine at each session (0.5 mg/kg)	35 mg	25 mg	35 mg

LEC-5 = Life Events Checklist for DSM-5; CTQ = Childhood Trauma Questionnaire; CGI-S = Clinical Global Impressions Scale—Severity; CAPS = Clinician-Administered PTSD Scale for DSM-5; * criteria for complex PTSD (ICD-11) fulfilled.

## Data Availability

Data supporting the results can be found in the Supplementary Material (Table S3). Here, we provide complete data.

## Supplementary Materials

The following supporting information can be downloaded at https://www.mdpi.com/article/10.3390/bs14080717/s1, Table S1: Psychological assessments and time points; Table S2: Side effects and other immediate effects; Table S3: Baseline, post- and follow-up assessment. References [40,41,42,43,44,45,46,47,48,49,50] are cited in the Supplementary Materials.
