# Supplementary material for: Combined Effects of Nasal Ketamine and Trauma-Focused Psychotherapy in Treatment-Resistant Post-Traumatic Stress Disorder: A Pilot Case Series
Source: Behav Sci (Basel). 2024 Aug 16;14(8):717. doi: 10.3390/bs14080717 (PMC11351759; doi:10.3390/bs14080717)
Supplement: Supplementary file 1 [file behavsci-14-00717-s001.zip › Supplemental Table S3 Baseline post and follow up assessment.pdf]

Supplementary Table S3

Baseline, post- and follow-up assessment

**Patient**	**1**			**2**			**3**		
**Time point**	**Pre**	**Post**	**Follow-up**	**Pre**	**Post**	**Follow-up**	**Pre**	**Post**	**Follow-up**
**CAPS-5_1**	4	3	2	2	2	2	2	1	0
**CAPS-5_1S**	yes	yes	yes	yes	yes	yes	yes	no	no
**CAPS-5_2**	4	4	4	0	1	0	0	0	0
**CAPS-5_2S**	yes	yes	yes	no	no	no	no	no	no
**CAPS-5_3**	4	3	2	2	0	1	0	0	0
**CAPS-5_3S**	yes	yes	yes	yes	no	no	no	no	no
**CAPS-5_4**	4	2	3	3	3	2	3	1	3
**CAPS-5_4S**	yes	yes	yes	yes	yes	yes	yes	no	yes
**CAPS-5_5**	4	4	3	3	3	2	1	0	0
**CAPS-5_5S**	yes	yes	yes	yes	yes	yes	no	no	no

**CAPS-5_6**	4	2	3	4	4	3	4	3	0
**CAPS-5_6S**	yes	yes	yes	yes	yes	yes	yes	yes	no
**CAPS-5_7**	4	3	2	3	0	2	4	2	0
**CAPS-5_7S**	yes	yes	yes	yes	no	yes	yes	yes	no
**CAPS-5_8**	4	3	2	0	2	1	4	1	2
**CAPS-5_8S**	yes	yes	yes	no	yes	no	yes	no	yes
**CAPS-5_9**	4	3	4	4	2	3	4	1	2
**CAPS-5_9S**	yes	yes	yes	yes	yes	yes	yes	no	yes
**CAPS-5_10**	4	4	2	3	3	2	4	1	1
**CAPS-5_10S**	yes	yes	yes	yes	yes	yes	yes	no	no
**CAPS-5_11**	4	3	2	3	4	3	4	1	0
**CAPS-5_11S**	yes	yes	yes	yes	yes	yes	yes	no	no
**CAPS-5_12**	2	3	3	3	1	1	4	2	1
**CAPS-5_12S**	yes	yes	yes	yes	no	no	yes	yes	no
**CAPS-5_13**	4	3	2	3	3	3	4	3	3
**CAPS-5_13S**	yes	yes	yes	yes	yes	yes	yes	yes	yes

**CAPS-5_14**	3	3	3	4	4	3	4	3	1
**CAPS-5_14S**	yes	yes	yes	yes	yes	yes	yes	yes	no
**CAPS-5_15**	0	0	1	0	0	0	4	2	2
**CAPS-5_15S**	no	no	no	no	no	no	yes	yes	yes
**CAPS-5_16**	2	1	3	1	0	0	0	0	0
**CAPS-5_16S**	yes	no	yes	no	no	no	no	no	no
**CAPS-5_17**	4	3	2	3	2	2	1	0	0
**CAPS-5_17S**	yes	yes	yes	yes	yes	yes	no	no	no
**CAPS-5_18**	4	3	3	3	4	1	3	0	0
**CAPS-5_18S**	yes	yes	yes	yes	yes	no	yes	no	no
**CAPS-5_19**	2	1	2	3	3	0	3	2	2
**CAPS-5_19S**	yes	no	yes	yes	yes	no	yes	yes	yes
**CAPS-5_20**	4	3	3	3	3	0	3	0	0
**CAPS-5_20S**	yes	yes	yes	yes	yes	no	yes	no	no
**CAPS-5_21**	no	no	no	no	no	no	yes	yes	yes
**CAPS-5_22**	yes	yes	yes	yes	yes	yes	yes	yes	yes

**CAPS-5_23**	4	2	2	3	3	2	3	2	2
**CAPS-5_23S**	yes	yes	yes	yes	yes	yes	yes	yes	yes
**CAPS-5_24**	3	3	3	3	3	3	3	3	0
**CAPS-5_24S**	yes	yes	yes	yes	yes	yes	yes	yes	no
**CAPS-5_25**	3	3	3	3	3	1	3	1	0
**CAPS-5_25S**	yes	yes	yes	yes	yes	no	yes	no	no
**CAPS-5_26**	1	1	1	1	1	1	1	1	1
**CAPS-5_27**	4	4	4	2-3	2	2	2	1	1
**CAPS-5_28**	N/A	3	4	N/A	3	M	N/A	3	2
**CAPS-5_29**	4	1	0	2	0	0	1	0	0
**CAPS-5_29S**	yes	no	no	yes	no	no	no	no	no
**CAPS-5_30**	3	2	3	0	0	0	0	1	1
**CAPS-5_30S**	yes	yes	yes	no	no	no	no	no	no
**CAPS_SUM**	69	54	51	50	44	31	56	23	17
**HAMD_1**	1	1	1	3	3	2	3	2	2
**HAMD_2**	3	2	2	3	2	1	2	2	2

**HAMD_3**	0	0	0	1	1	1	2	1	1
**HAMD_4**	2	1	2	2	2	0	0	0	0
**HAMD_5**	2	2	2	2	2	0	2	0	0
**HAMD_6**	2	0	2	2	1	0	2	0	0
**HAMD_7**	1	1	1	3	2	1	1	1	0
**HAMD_8**	0	0	0	1	0	0	2	0	0
**HAMD_9**	1	0	1	0	0	0	0	0	0
**HAMD_10**	4	2	4	3	3	3	4	1	1
**HAMD_11**	3	1	2	3	2	2	2	1	0
**HAMD_12**	1	1	1	0	0	1	2	0	0
**HAMD_13**	0	1	1	0	2	1	1	1	0
**HAMD_14**	1	1	1	0	1	1	0	0	0
**HAMD_15**	0	0	0	0	0	0	1	0	1
**HAMD_16a**	0	0	0	0	0	1	0	0	0
**HAMD_16b**	0	0	0	0	0	0	0	0	0
**HAMD_17**	0	0	0	0	0	0	0	0	0

**HAMD_18a**	0	0	0	0	0	0	0	1	1
**HAMD_18b**	0	0	0	0	0	0	0	1	1
**HAMD_19**	4	0	0	1	0	0	1	1	1
**HAMD_20**	1	0	1	0	0	1	2	1	0
**HAMD_21**	0	0	0	0	0	0	0	0	0
**HAMD_SUM**	21	13	20	23	21	14	24	9	7
**CGI_S**	6	6	6	5	5	4	6	4	3
**CGI_I**	-	3	4	-	3	3	-	3	2
**CTQ_1**	3			1			1		
**CTQ_2**	3			3			2		
**CTQ_3**	4			5			5		
**CTQ_4**	1			1			4		
**CTQ_5**	2			2			1		
**CTQ_6**	4			1			3		
**CTQ_7**	2			3			3		
**CTQ_8**	5			3			4		

**CTQ_9**	4	1	1
**CTQ_10**	1	2	1
**CTQ_11**	3	4	1
**CTQ_12**	3	4	3
**CTQ_13**	1	3	3
**CTQ_14**	4	5	5
**CTQ_15**	5	4	3
**CTQ_16**	1	2	1
**CTQ_17**	3	1	1
**CTQ_18**	5	3	2
**CTQ_19**	1	2	2
**CTQ_20**	3	1	3
**CTQ_21**	1	1	1
**CTQ_22**	1	2	1
**CTQ_23**	3	1	1
**CTQ_24**	3	2	3

**CTQ_25**	5	4	4
**CTQ_26**	1	5	3
**CTQ_27**	4	1	3
**CTQ_28**	1	3	2
**LEC-5_1**	6	3	5
**LEC-5_2**	6	2	5
**LEC-5_3**	6	2 & 3	3
**LEC-5_4**	6	5	5
**LEC-5_5**	1	3	5
**LEC-5_6**	1	1 & 2 & 3	2
**LEC-5_7**	1	3	5
**LEC-5_8**	1	3	5
**LEC-5_9**	1	1 & 3	1
**LEC-5_10**	6	3	3
**LEC-5_11**	6	5	5
**LEC-5_12**	1	3	3

**LEC-5_13**	1			2, 3			1		
**LEC-5_14**	6			3			3		
**LEC-5_15**	2			5			3		
**LEC-5_16**	6			6			5		
**LEC-5_17**	1			1			1		
**DERS_1**	1	4	2	2	2	1	2	4	2
**DERS_2**	2	4	2	2	1	1	2	3	3
**DERS_3**	4	3	3	4	3	4	3	4	4
**DERS_4**	5	2	3	4	4	5	3	1	3
**DERS_5**	5	2	3	4	4	5	5	2	4
**DERS_6**	2	4	2	2	2	2	2	3	2
**DERS_7**	2	4	2	1	1	1	3	4	3
**DERS_8**	2	4	3	3	3	3	2	3	3
**DERS_9**	4	2	3	5	4	5	5	2	4
**DERS_10**	2	3	2	4	5	5	2	1	2
**DERS_11**	5	5	4	5	5	5	4	5	4

**DERS_12**	4	4	4	2	4	4	5	4	4
**DERS_13**	2	4	4	4	5	5	5	4	4
**DERS_14**	4	4	4	1	4	2	5	4	4
**DERS_15**	5	2	4	5	5	5	5	4	4
**DERS_16**	5	3	4	5	5	5	5	4	5
**DERS_17**	1	3	3	5	2	5	2	4	3
**DERS_18**	5	4	4	5	5	5	5	5	5
**DERS_19**	5	4	4	5	5	1	5	4	5
**DERS_20**	1	3	2	3	2	2	5	3	2
**DERS_21**	1	4	2	2	4	5	2	4	5
**DERS_22**	4	4	3	1	1	3	1	3	2
**DERS_23**	5	4	4	5	5	5	5	2	5
**DERS_24**	2	4	2	2	2	4	2	2	2
**DERS_25**	2	4	4	5	4	4	4	4	4
**DERS_26**	5	4	4	5	5	5	5	5	5
**DERS_27**	5	4	4	5	1	1	4	5	4

**DERS_28**	4	3	3	5	5	5	5	5	4
**DERS_29**	5	3	4	4	4	4	5	5	5
**DERS_30**	5	3	4	5	5	5	4	5	4
**DERS_31**	5	3	3	5	5	5	5	5	4
**DERS_32**	4	2	4	2	2	1	4	5	4
**DERS_33**	5	3	4	5	5	5	5	5	5
**DERS_34**	2	3	3	1	1	2	1	2	2
**DERS_35**	3	3	3	5	5	5	5	5	5
**DERS_36**	5	3	4	5	5	5	5	5	5
**DERS_SUM**	152	108	131	148	153	144	154	137	148
**STAI_1**	1	2	3	2	2	2	2	1	1
**STAI_2**	1	1	2	1	1	2	1	1	1
**STAI_3**	3	2	3	3	3	3	4	3	4
**STAI_4**	3	2	2	4	3	4	4	4	4
**STAI_5**	1	2	1	1	2	1	1	1	1
**STAI_6**	3	3	2	2	3	2	2	2	3

**STAI_7**	3	3	3	3	4	4	4	4	4
**STAI_8**	1	1	2	2	3	1	1	1	1
**STAI_9**	3	3	2	4	2	4	4	3	4
**STAI_10**	2	2	2	1	2	2	1	1	1
**STAI_11**	1	1	2	1	2	2	1	1	4
**STAI_12**	3	2	2	3	3	3	3	2	3
**STAI_13**	2	1	2	3	3	3	2	2	2
**STAI_14**	2	2	2	3	2	4	4	3	3
**STAI_15**	3	1	2	1	1	2	1	1	1
**STAI_16**	2	2	2	1	2	2	1	1	1
**STAI_17**	2	2	2	4	3	4	4	4	4
**STAI_18**	1	1	1	2	1	2	4	4	4
**STAI_19**	2	2	2	1	1	1	1	1	1
**STAI_20**	2	1	2	1	1	1	1	1	1
**STAI_21**	2	2	2	1	1	1	1	1	1
**STAI_22**	3	3	3	4	4	3	4	4	4

**STAI_23**	2	2	2	3	1	2	4	4	3
**STAI_24**	2	1	2	4	3	4	4	4	4
**STAI_25**	2	1	2	2	2	3	2	2	2
**STAI_26**	1	3	2	1	1	1	1	1	1
**STAI_27**	1	2	2	1	2	1	1	1	1
**STAI_28**	2	2	3	3	4	4	4	4	4
**STAI_29**	2	1	3	3	3	4	4	4	4
**STAI_30**	2	2	2	1	1	1	1	1	1
**STAI_31**	3	2	2	3	3	4	4	4	4
**STAI_32**	3	2	3	3	2	3	4	4	4
**STAI_33**	2	2	2	1	1	1	1	1	1
**STAI_34**	3	2	3	4	4	4	4	3	3
**STAI_35**	3	2	2	4	3	4	4	4	4
**STAI_36**	2	3	2	1	2	1	1	1	1
**STAI_37**	3	1	3	3	4	4	4	3	4
**STAI_38**	3	1	3	3	2	3	4	4	4

**STAI_39**	2	2	2	1	2	1	1	1	1
**STAI_40**	3	3	3	4	4	4	4	4	4
**STAI_STATE_SUM**	59	56	51	69	60	67	74	71	72
**STAI_TRAIT_SUM**	57	42	55	71	64	74	78	76	76
**GSE_1**	3	3	3	3	3	3	3	3	3
**GSE _2**	3	4	4	3	3	3	3	3	2
**GSE _3**	2	3	2	2	2	1	1	2	2
**GSE _4**	2	3	2	2	2	2	2	3	2
**GSE _5**	2	2	2	2	2	2	2	3	2
**GSE _6**	2	2	2	2	1	2	2	2	2
**GSE _7**	3	3	3	3	3	3	3	3	3
**GSE _8**	3	3	3	3	2	2	2	3	3
**GSE _9**	2	2	3	3	2	2	2	3	2
**GSE _10**	2	2	2	3	3	3	3	3	3
**GSE _SUM**	24	27	26	26	23	23	23	28	24
**Shut-D_1**	1	0	1	0	0	0	0	0	0

**Shut-D_2**	3	1	3	1	2	2	0	2	2
**Shut-D_3**	2	0	0	1	1	0	0	0	0
**Shut-D_4**	3	1	0	1	0	0	0	1	1
**Shut-D_5**	3	1	1	3	1	0	0	0	0
**Shut-D_6**	2	0	0	0	0	0	0	0	0
**Shut-D_7**	2	0	0	1	1	0	0	1	0
**Shut-D_8**	0	0	0	2	2	1	0	3	2
**Shut-D_9**	0	0	0	1	0	0	0	0	0
**Shut-D_10**	2	2	3	3	2	2	1	1	2
**Shut-D_11**	1	0	0	2	0	1	0	0	0
**Shut-D_12**	2	3	2	0	0	0	0	0	0
**Shut-D_13**	0	0	0	0	0	0	0	0	0
**Shut-D_SUM**	21	8	10	15	9	6	1	8	7
**ITQ_0**	f	f	f	d	d	d	e	e	3
**ITQ_P1**	4	4	3	1	1	0	0	0	0
**ITQ_P2**	4	2	2	3	2	1	2	0	0

**ITQ_P3**	4	3	4	4	3	3	4	3	3
**ITQ_P4**	4	3	3	4	1	4	3	3	3
**ITQ_P5**	4	2	2	4	4	4	0	2	0
**ITQ_P6**	4	2	3	4	4	4	2	2	2
**ITQ_P7**	4	2	4	3	4	3	1	4	0
**ITQ_P8**	4	2	4	3	3	4	1	3	0
**ITQ_P9**	4	2	3	4	4	4	0	3	0
**ITQ_C1**	4	2	3	4	3	4	4	4	4
**ITQ_C2**	4	2	3	4	4	4	4	4	4
**ITQ_C3**	2	1	2	3	3	3	4	4	4
**ITQ_C4**	3	1	2	3	3	3	4	4	4
**ITQ_C5**	4	2	2	4	2	4	4	4	4
**ITQ_C6**	4	1	4	4	4	4	4	4	4
**ITQ_C7**	4	2	3	3	4	4	4	4	4
**ITQ_C8**	3	2	4	4	4	4	4	3	4
**ITQ_C9**	4	2	3	4	4	4	4	3	4

**PTSD yes/no**	yes	yes	yes	yes	yes	no	yes	no	no
**PTSD_SUM**	24	16	17	20	15	16	11	10	8
**DSO yes/no**	yes	yes	yes	yes	yes	yes	yes	yes	yes
**DSO_SUM**	21	9	16	22	19	22	24	24	24

List of abbreviations

CAPS-5 Clinician-Administered PTSD Scale for DSM-5

CAPS-5 S Clinician-Administered PTSD Scale for DSM-5 Item Summary

CAPS_SUM Clinician-Administered PTSD Scale for DSM-5 severity of symptoms sum score

HAMD Hamilton-Depression Rating Scale

HAMD_SUM Hamilton-Depression Rating Scale Sum Score

CGI S Clinical Global Impressions Scale-Severity

CGI I Clinical Global Impressions Scale-Improvement

CTQ Childhood Trauma Questionnaire

LEC-5 Life Events Checklist for DSM-5

DERS Difficulties in Emotion Regulation Scale

DERS_SUM Difficulties in Emotion Regulation Scale Sum Score

STAI The State-Trait Anxiety Inventory

STAI_STATE_SUM State Anxiety Sum Score

STAI_TRAIT_SUM Trait Anxiety Sum Score

GSE General Self-Efficacy Scale

GSE _SUM Sum Score General Self-Efficacy Scale

Shut-D Shutdown Dissociation Scale

Shut-D_SUM Shutdown Dissociation Scale Sum Score

ITQ International Trauma Questionnaire

PTSD Post traumatic stress disorder

DSO Disturbances in Self-Organization

N/A Not Applicable

M Missing
