# Supplementary material for: Combined Effects of Nasal Ketamine and Trauma-Focused Psychotherapy in Treatment-Resistant Post-Traumatic Stress Disorder: A Pilot Case Series
Source: Behav Sci (Basel). 2024 Aug 16;14(8):717. doi: 10.3390/bs14080717 (PMC11351759; doi:10.3390/bs14080717)
Supplement: Supplementary file 1 [file behavsci-14-00717-s001.zip › Supplementary Table S1 Psychological assessments and time points.pdf]

Supplementary Table S1

Psychological assessments and time points

**Type**	**Name of instrument**	**Description**	**Baseline**	**Post**	**Follow-up**
**Clinician-rated**	Clinician-Administered PTSD Scale for DSM-5, CAPS-5 (Weathers et al., 2015)	Consists of 30 items assessing symptoms, existence, and severity of PTSD. The first 20 items offer a choice of five possible answers (0-4). A higher total score, ranging between 0-80, indicates a greater severity of PTSD-associated symptoms. A minimum score in each cluster is necessary for a positive diagnosis.	x	x	x
	Hamilton-Depression Rating Scale, HAMD (Hamilton, 1960)	Consists of 21 items with answers on five-point or three-point scales (0-2; 0-4). Items 1-17 measure depression severity. Higher scores signify more severe symptoms (0-8: no depression, 9-16: mild depression, 17-24: moderate	x	x	x

		depression, > 24: severe depression).			
	Clinical Global Impressions Scale-Severity, CGI-S (Guy, 1976)	Assesses the severity of mental illness at the time of examination on a scale of one to seven (1 = normal/not ill – 7 = extremely ill).	x	x	x
	Clinical Global Impressions Scale-Improvement, CGI-I (Guy, 1976)	Assesses the degree of improvement or worsening of the patient's illness relative to the beginning of the intervention on a scale of one to seven (1 = Very much improved – 7 = Very much worse).		x	x
**Self-rating**	Childhood Trauma Questionnaire, CTQ (Bernstein et al., 2003)	Consists of 28 items with five possible answers indicating the frequency of experiences of childhood abuse and neglect (1 = never true – 5 = very often true), including physical, emotional, and sexual abuse, and physical and emotional neglect. The total score ranges from 25 to 125, with	x		

		higher scores indicating more severe abuse or neglect.			
	Life Events Checklist for DSM-5, LEC-5 (Weathers et al., 2013)	Consists of a list of 17 potentially traumatic events assessing their occurrence during the patient's lifespan in addition to the circumstances of the events (e.g., "happened to me"; "witnessed it").	x		
	International Trauma Questionnaire, ITQ (Cloitre et al., 2018)	Consists of 18 items with five possible answers assessing the extent to which a patient has been bothered by trauma-associated experiences (0 = not at all - 4 = extremely). The first nine items explore the core symptoms of PTSD, while the additional nine items explore symptoms related to complex PTSD according to ICD-11. A higher score, with a required minimum in each cluster, is associated with more severe symptoms.	x	x	x

	Difficulties in Emotion Regulation Scale, DERS (Gratz & Roemer, 2004)	Consists of 36 items with five possible answers assessing the patient's difficulties in regulating their emotions. A higher overall score, ranging between 36-180, indicates higher difficulties, whereas a score of 77.99 ± 20.72 is considered normal for females.	x	x	x
	The State-Trait Anxiety Inventory, STAI (Spielberger et al., 1983)	Consists of 40 items with four possible answers. Items 1-20 explore State Anxiety (current experience of anxiety), whereas items 21-40 explore Trait Anxiety (general experience of anxiety). The State and Trait subscores range from 20 – 80, with a higher score indicating more severe anxiety.	x	x	x
	General Self-Efficacy Scale, GSE (Tipton & Worthington, 1984)	Consists of 10 items with four possible answers assessing the extent of an individual's belief in their ability to deal with life challenges (1 = not at all true - 4 =	x	x	x

		exactly true). The sum score ranges from 10-40, with a higher score indicating higher self-belief.			
	Shutdown Dissociation Scale, Shut-D (Schalinski et al., 2015, 2016)	Consists of 13 items with four possible answers assessing the extent to which a range of dissociative symptoms have been experienced since a traumatic event or in the past six months (0 = not at all - 3 = 5 or more times a week). Summed scores range from 0 to 39. A total score ≥ 16 indicates a dissociative subtype of PTSD according to DSM-5.	x	x	x

References

Bernstein, D. P., Stein, J. A., Newcomb, M. D., Walker, E., Pogge, D., Ahluvalia, T., Stokes, J., Handelsman, L., Medrano, M., & Desmond, D. (2003). Development and validation of a brief screening version of the Childhood Trauma Questionnaire. *Child Abuse & Neglect*, *27*(2), 169–190.

Cloitre, M., Shevlin, M., Brewin, C. R., Bisson, J. I., Roberts, N. P., Maercker, A., Karatzias, T., & Hyland, P. (2018). The International Trauma Questionnaire: Development of a self-

report measure of ICD-11 PTSD and complex PTSD. *Acta Psychiatrica Scandinavica*, *138*(6), 536–546.

Gratz, K. L., & Roemer, L. (2004). Multidimensional assessment of emotion regulation and dysregulation: Development, factor structure, and initial validation of the difficulties in emotion regulation scale. *Journal of Psychopathology and Behavioral Assessment*, *26*, 41–54.

Guy, W. (1976). Clinical global impressions scale. *Psychiatry*.

Hamilton, M. (1960). A rating scale for depression. *Journal of Neurology, Neurosurgery, and Psychiatry*, *23*(1), 56.

Schalinski, I., Schauer, M., & Elbert, T. (2015). The shutdown dissociation scale (Shut-D). *European Journal of Psychotraumatology*, *6*(1), 25652.

Schalinski, I., Schauer, M., & Elbert, T. (2016). *Shut-D - Shutdown-Dissoziationsskala*. https://psycharchives.org/en/item/5c519cd7-ebb2-4a22-850f-b3d1ca25bddf

Spielberger, C., Gorsuch, R., Lushene, R., Vagg, P., & Jacobs, G. (1983). Manual for the State-trait anxiety inventory (form Y): Self-evaluation questionnairepress in Consulting Psychologists. *California: Palo Alto*, 1–36.

Tipton, R. M., & Worthington, E. L. (1984). The measurement of generalized self-efficacy: A study of construct validity. *Journal of Personality Assessment*.

Weathers, F. W., Blake, D. D., Schnurr, P. P., Kaloupek, D. G., Marx, B. P., & Keane, T. M. (2013). *The life events checklist for DSM-5 (LEC-5)*.

Weathers, F. W., Bovin, M. J., Lee, D. J., Sloan, D. M., Schnurr, P. P., Kaloupek, D. G., Keane, T. M., & Marx, B. P. (2015). Clinician-administered ptsd scale for DSM-5. *Psychological Assessment*.
