# Supplementary material for: Combined Effects of Nasal Ketamine and Trauma-Focused Psychotherapy in Treatment-Resistant Post-Traumatic Stress Disorder: A Pilot Case Series
Source: Behav Sci (Basel). 2024 Aug 16;14(8):717. doi: 10.3390/bs14080717 (PMC11351759; doi:10.3390/bs14080717)
Supplement: Supplementary file 1 [file behavsci-14-00717-s001.zip › Supplementary Table S2 Side effects and other immediate effects.pdf]

Supplementary Table S2

Side effects and other immediate effects

**Patient 1**		**Ketamine session**							
		**1**	**2**	**3**	**4**	**5**	**6**	**7**	**8**
**Nausea**	pre	1	4	4	2	1	1	1	1
	post	2	1	1	2	1	1	1	1
	change score	1	-3	-3	0	0	0	0	0
**Tiredness**	pre	7	8	7	4	5	4	3	2
	post	8	5	8	6	1	2	3	7
	change score	1	-3	1	2	-4	-2	0	5
**Dizziness**	pre	6	1	6	2	3	2	4	2
	post	6	4	6	7	2	1	3	2
	change score	0	3	0	5	-1	-1	-1	0
**Headache**	pre	1	1	1	1	1	2	1	1
	post	1	1	1	1	1	1	1	2
	change score	0	0	0	0	0	-1	0	1
**Restlessness**	pre	8	3	6	5	5	6	5	4
	post	2	2	2	6	3	4	2	3
	change score	-6	-1	-4	1	-2	-2	-3	-1
**Anxiety**	pre	9	2	9	5	3	3	4	3
	post	2	2	2	2	1	6	1	1
	change score	-7	0	-7	-3	-2	3	-3	-2
**Tension**	pre	9	2	6	5	6	8	4	5
	post	1	2	2	2	3	6	3	2

	change score	-8	0	-4	-3	-3	-2	-1	-3
**Happiness**	pre	5	5	4	4	3	3	4	5
	post	6	5	4	4	3	3	7	4
	change score	1	0	0	0	0	0	3	-1
**Intrusions**	pre	7	5	9	3	4	5	4	1
	post	1	2	1	1	1	8	1	1
	change score	-6	-3	-8	-2	-3	3	-3	0
**Body (part) feels numb**	pre	8	1	1	1	1	10	4	1
	post	1	1	2	2	2	1	2	1
	change score	-7	0	1	1	1	-9	-2	0
**body feels weak and warm**	pre	1	1	1	1	1	1	1	1
	post	8	2	1	1	1	1	1	1
	change score	7	1	0	0	0	0	0	0
**Derealisation/depersonalisation**	pre	3	1	2	1	1	1	3	1
	post	3	3	2	2	1	6	1	2
	change score	0	2	0	1	0	5	-2	1
**feeling of not hearing or seeing properly**	pre	5	2	2	1	1	3	1	1
	post	4	1	3	2	1	1	1	1
	change score	-1	-1	1	1	0	-2	0	0
**feeling energetic**	pre	3	4	4	3	5	4	7	6
	post	1	5	5	4	6	7	7	3

	change score	-2	1	1	1	1	3	0	-3
**Hallucinations**	pre	1	1	1	1	1	1	1	1
	post	1	1	1	1	1	1	1	1
	change score	0	0	0	0	0	0	0	0
**Patient 2**									
		1	2	3	4	5	6	7	8
**Nausea**	pre	4	2	5	4	3	3	4	1
	post	1	1	1	3	1	1	1	1
	change score	-3	-1	-4	-1	-2	-2	-3	0
**Tiredness**	pre	8	7	8	7	8	7	9	8
	post	6	8	9	4	6	9	3	9
	change score	-2	1	1	-3	-2	2	-6	1
**Dizziness**	pre	3	2	3	1	1	2	4	2
	post	1	1	2	1	2	2	1	1
	change score	-2	-1	-1	0	1	0	-3	-1
**Headache**	pre	1	2	4	4	5	3	2	5
	post	1	1	1	1	1	1	1	1
	change score	0	-1	-3	-3	-4	-2	-1	-4
**Restlessness**	pre	9	9	9	9	9	6	9	8
	post	2	2	8	3	6	4	3	5
	change score	-7	-7	-1	-6	-3	-2	-6	-3
**Anxiety**	pre	8	7	7	8	9	8	9	9
	post	1	1	7	1	4	3	2	2
	change score	-7	-6	0	-7	-5	-5	-7	-7

**Tension**	pre	9	9	9	9	9	8	10	9
	post	2	2	9	1	5	4	3	5
	change score	-7	-7	0	-8	-4	-4	-7	-4
**Happiness**	pre	2	2	3	3	4	4	2	1
	post	3	2	2	4	5	2	3	1
	change score	1	0	-1	1	1	-2	1	0
**Intrusions**	pre	7	3	6	5	4	2	6	7
	post	1	1	1	1	1	2	1	1
	change score	-6	-2	-5	-4	-3	0	-5	-6
**Body (part) feels numb**	pre	1	1	1	1	1	1	1	1
	post	2	3	2	3	6	3	3	1
	change score	1	2	1	2	5	2	2	0
**body feels weak and warm**	pre	1	1	1	1	1	1	1	1
	post	2	3	3	6	8	1	3	2
	change score	1	2	2	5	7	0	2	1
**Derealisation/depersonalisation**	pre	1	1	1	1	1	1	1	1
	post	1	1	1	1	1	1	1	1
	change score	0	0	0	0	0	0	0	0
**feeling of not hearing or seeing properly**	pre	2	1	1	3	1	2	2	1
	post	3	1	2	7	2	5	7	5
	change score	1	0	1	4	1	3	5	4
**feeling energetic**	pre	2	1	1	2	5	2	1	2
	post	3	4	1	6	4	3	1	3
	change score	1	3	0	4	-1	1	0	1

**Hallucinations**	pre	1	1	1	1	1	1	1	1
	post	1	1	1	1	1	1	1	1
	change score	0	0	0	0	0	0	0	0
**Patient 3**									
		1	2	3	4	5	6	7	8
**Nausea**	pre	5	4	7	1	1	1	1	1
	post	1	9	6	1	1	1	1	1
	change score	-4	5	-1	0	0	0	0	0
**Tiredness**	pre	10	8	9	8	8	8	8	9
	post	6	8	5	8	9	8	8	8
	change score	-4	0	-4	0	1	0	0	-1
**Dizziness**	pre	3	4	7	5	4	5	5	5
	post	5	9	6	6	5	5	7	6
	change score	2	5	-1	1	1	0	2	1
**Headache**	pre	2	3	8	5	6	3	4	5
	post	1	3	6	4	4	2	2	3
	change score	-1	0	-2	-1	-2	-1	-2	-2
**Restlessness**	pre	8	7	4	4	4	3	4	4
	post	2	2	1	1	1	1	1	1
	change score	-6	-5	-3	-3	-3	-2	-3	-3
**Anxiety**	pre	9	7	3	4	4	3	4	4
	post	2	5	1	1	2	3	1	1
	change score	-7	-2	-2	-3	-2	0	-3	-3
**Tension**	pre	9	7	3	3	4	3	4	4

	post	1	4	2	1	1	1	1	1
	change score	-8	-3	-1	-2	-3	-2	-3	-3
**Happiness**	pre	2	1	1	2	2	2	2	2
	post	1	1	2	2	2	2	2	2
	change score	-1	0	1	0	0	0	0	0
**Intrusions**	pre	3	4	3	2	3	3	3	4
	post	2	1	3	1	1	1	1	4
	change score	-1	-3	0	-1	-2	-2	-2	0
**Body (part) feels**	pre	1	1	1	1	1	1	1	1
**numb**	post	1	3	1	2	1	2	1	3
**body feels weak**	change score	0	2	0	1	0	1	0	2
**and warm**	pre	1	1	1	1	1	1	1	1
	post	3	4	1	1	1	1	1	1
	change score	2	3	0	0	0	0	0	0
**Derealisation/dep**	pre	7	3	5	3	1	4	4	3
**ersonalisation**	post	1	2	1	1	1	1	1	1
	change score	-6	-1	-4	-2	0	-3	-3	-2
**feeling of not**	pre	1	3	1	1	1	1	1	1
**hearing or seeing**	post	4	4	1	3	1	1	1	1
**properly**	change score	3	1	0	2	0	0	0	0
**feeling energetic**	pre	1	1	1	1	1	1	1	1
	post	3	1	2	1	1	1	1	1
	change score	2	0	1	0	0	0	0	0
**Hallucinations**	pre	1	1	1	1	1	1	1	1

	post	1	1	1	1	1	1	1	1
	change score	0	0	0	0	0	0	0	0
